# Working Alliance in Patients with Severe Mental Illness Who Need a Crisis Intervention Plan

**DOI:** 10.1007/s10597-015-9839-7

**Published:** 2015-02-21

**Authors:** Asia Ruchlewska, Astrid M. Kamperman, Mark van der Gaag, André I. Wierdsma, Niels C. L. Mulder

**Affiliations:** 1 Department of Psychiatry, ESPRi Epidemiological and Social Psychiatric Research Institute, Erasmus University Medical Centre, ‘s Gravendijkwal 230, 3015 CE Rotterdam, The Netherlands; 2 Parnassia Psychiatric Institute, The Hague, The Netherlands; 3 VU University and EMGO Institute for Health and Care Research, Amsterdam, The Netherlands; 4 BavoEuropoort, Rotterdam, The Netherlands

**Keywords:** Working alliance, Therapist patient relationship, Severe mental illness, Psychosis, Prediction

## Abstract

Working alliance has been characterized as an important predictor of positive treatment outcomes. We examined whether illness insight, psychosocial functioning, social support and locus of control were associated with working alliance as perceived by both patient and clinician. We assessed 195 outpatients with psychotic or bipolar disorders. Our findings indicated that patients rated the alliance more positively when they experienced a greater need for treatment, fewer behavioral and social problems, and more psychiatric symptoms. Clinicians rated the alliance more positively in patients who reported fewer social problems and better illness insight. Patients’ demographic characteristics, including being female and married, were also positively related to the clinician-rated alliance. Our results suggest that patients and clinicians have divergent perceptions of the alliance. Clinicians may need help developing awareness of the goals and tasks of patients with certain characteristics, i.e., singles, men, those with poor illness insight and those who report poor social functioning.

## Introduction

A good working alliance has been characterized as an important predictor of positive outcomes for a number of treatments (Horvath and Symonds 1991; Martin et al. 2000). However, due to factors such as poor insight into their illness and into the need for treatment, some patients with schizophrenia and their clinicians find it difficult to form a therapeutic relationship (Frank and Gunderson 1990; Wittorf et al. 2009).

As one might expect, the severity of symptoms and subsequent impairments has been found to be associated with working alliance in patients with severe mental illness. However, the results vary widely, and may relate to the patient’s and clinician’s perceptions of their working alliance, or to the concordance between these perceptions. To start with the latter, Lysaker et al. (2011) found a higher level of concordance between patients and clinicians in patients who experienced more negative symptoms and more impairments (Lysaker et al. 2011). On the other hand, Davis and Lysaker (2004) found that while more impaired patients reported better alliances, their clinicians appraised the alliances more negatively. And while other authors (Barrowclough et al. 2010; Couture et al. 2006; Wittorf et al. 2009) have also reported a negative association between clinician-rated working alliance and the presence and severity of symptoms, Barrowclough et al. (2010) found an opposite association: that clinicians rated the working alliance more positively in patients with higher levels of self-reported depression. Beardsmore and Emseley found no association between symptom severity and alliance. Finally, McCabe and Priebe (2003) reported that patients with more severe symptoms gave a poorer rating to their working alliance.

Although some researchers suggest that patients who perceive their working alliance more positively do so because they have a better understanding of their illness (Barrowclough et al. 2010; Wittorf et al. 2009), the mechanism behind this relationship remains unclear. Patients who rated their working relationship highly have also been found to have a positive attitude towards medication and towards living with family (Barrowclough et al. 2010; Couture et al. 2006; Wittorf et al. 2009).

In patients with psychotic or bipolar disorders, we showed that the clinicians’ perspective on the quality of the working alliance was an important predictor of whether a treatment capable of preventing psychiatric crises had been properly implemented (Ruchlewska et al., submitted). This was shown in an RCT designed to examine the effects of a crisis plan—a particular type of advance statement developed in Dutch psychiatric care (Ruchlewska et al. 2009). Advance statements are used with patients with psychiatric disorders to document the treatment they would prefer if faced with a future mental health crisis or period of incapacity. The term “psychiatric advance statement” encompasses a range of instruments used in psychiatric care, such as psychiatric advance directives, wellness recovery action plans, and joint crisis plans. These vary in form, content, and judicial context (Henderson et al. 2008). A crisis plan describes crisis prevention and contains practical information on the action to be taken in future psychiatric emergencies. The information is summarized on a small card—the ‘crisis card’—which users carry with them at all times. While crisis plans are developed on a voluntary basis and are not legally binding, they are important instruments for helping patients and clinicians to find mutual agreement on how to handle crisis situations. Although they may thus be important to preventing involuntary admissions (Henderson et al. 2004; Ruchlewska et al. 2014), little is known about the determinants of a good working alliance in the patient population that most needs one.

In the present cross-sectional study, which was part of the RCT referred to above, we tested two hypotheses. The first was that a higher level of psychosocial functioning—i.e., greater insight, fewer symptoms, better social functioning, fewer behavioral problems and more social support—would be associated with a clinician’s and patient’s perception of a better working alliance. The second was that the working alliance achieved with patients with an external locus of control—i.e., those who experience little control about forces that impact their lives—would be perceived more poorly by clinician and patient alike.

## Methods

### Setting

For this study we used the baseline data from a randomized controlled trial on the effects of crisis plans (Ruchlewska et al. 2009). We recruited patients from twelve Community Mental Health Teams at three mental-health institutions in Rotterdam, the Netherlands. As is usually the case in Dutch psychiatric care, these teams provide care to adult patients (>18 years) with serious and persistent mental illness—usually a psychotic, bipolar, or major depressive disorder (with or without co-morbid substance disorder) who also have psychosocial problems in multiple domains of life. Outpatient care ranges from office-based community psychiatric care, to more intensive assertive outreach treatment. Team case load is generally small (i.e., less than 350 patients). Rotterdam’s community mental-health care institution has a catchment area of approximately 1.3 million inhabitants. Costs are covered through national health insurance.

### Participants

On the basis of the inclusion and exclusion criteria formulated in the context of the RCT, the clinician and the researcher selected candidate participants from the clinicians’ caseloads. The patients who had been selected received an information letter about the study from their clinicians, who also requested permission for an independent researcher to contact them. After the study had been described in full, written informed consent was obtained. Participants received EUR 10 for each interview. We selected 537 patients, 212 of whom (40 %) were included in the study. After explanation of the research goals, 151 patients (28 %) refused to be contacted by the researcher or refused to participate in the study, and 174 (32 %) could not be contacted after several unsuccessful attempts [for details of recruitment and inclusion, see Ruchlewska et al. (2014)]. The design and implementation of this study were approved by the Dutch Union of Medical Ethics Trial Committees for Mental Health Organizations.

### Measures

#### Working Alliance Inventory (WAI)

To measure the quality of the working alliance from the patients’ and clinicians’ perspectives, we used the Dutch version of the Working Alliance Inventory (WAI) (Horvath and Greenberg 1989; Vervaeke and Vertommen 1996). This 36-item scale, which was rated on a 5-point scale, from 1 (‘no, I strongly disagree’) to 5 (‘yes, I strongly agree’), concerns three aspects of the therapeutic relationship: (1) tasks, i.e., the extent to which patient and therapist view the treatment tasks as relevant; (2) bonds, i.e., the personal attachment between the patient and clinician, which is created through trust, empathy and respect; and (3) goals, i.e., mutual agreement on and valuing of the outcomes of therapy. Higher scores indicate greater satisfaction with the alliance. The reliability of the WAI was measured using Cronbach’s alpha. In our sample, the patient’s and clinician’s instruments of the WAI both showed high levels of internal consistency (alpha = 0.94; alpha = 0.92), the range being 80–180 for the patients’ scale and 73–178 for the clinicians’ scale.

#### Psychosocial Functioning

Psychosocial functioning was assessed by an independent interviewer using the Dutch version of the 12-item Health of the Nation Outcome Scales (HoNOS) (Mulder et al. 2004; Wing et al. 1998). The HoNOS was completed by the researcher after a structured interview that quantified the psychosocial problems encountered within the previous 2 weeks. The items are rated from 0 (no problem) to 4 (severe to very severe problem). The intra-class correlation coefficient of the HoNOS total scores was 0.87 in the similar population, which indicates very good reliability (Wing et al. 1998). The range for this scale in our sample was a minimum of 1 and a maximum of 25. Four subscales concern behavioral problems (range in sample: 0–6), impairment (range in sample: 0–6), psychiatric symptoms (range in sample: 0–9) and social dysfunction (range in sample: 0–10).

#### Insight

Insight was assessed using a self-report Insight into Psychosis scale (Birchwood et al. 1994). This consists of eight statements to which the participant responds in one of three ways: agree, disagree and unsure. The three subscales concern the relabeling of symptoms, awareness of illness, and the perceived need for treatment. Higher scores suggest greater insight. The reliability for the total scale in our sample was high, with a Cronbach’s alpha of 0.75. The test–retest correlation was 0.90, indicating high reliability (Birchwood et al. 1994). Due to the non-normal distribution of this variable, the scores of the scale were log transformed.

#### Social Support

Social support was measured using the Adult Social Report scale (Ruehlman et al. 1999), a self-report scale comprising fourteen items that measure the respondent’s opinion of the help received from family and friends. Each item is rated on a 5-point scale from ‘no help at all’ to ‘very much help’. In our sample, the test–retest coefficient for this scale was 0.82, which indicates high reliability. The range of the scale was 15–61.

#### Locus of Control

Patients’ personal feeling of control over the forces impacting their lives was measured using MASTERY, a 7-item scale (Pearlin and Schooler 1978) in which each item is a statement reflecting the respondent’s perception of self. Four responses are rated from 1 (‘strongly disagree’) to 4 (‘strongly agree’). Higher scores indicated an external locus of control, meaning less control over the forces that impact the patient’s life. As measured by Cronbach’s alpha, the reliability of the Dutch version of this scale was 0.79 (Kempen 1992). The range of this scale in our sample was 5–25.

### Statistical Analysis

The data were checked for normality, and relationships between predictor variables were checked for collinearity. Correlations and differences between patients’ and clinicians’ working-alliance ratings were then examined using the Pearson correlations and paired *t* tests. Finally, backward multiple linear regression analysis was used to identify independent predictors of patient- and clinician-rated working alliances.

## Results

The questionnaire on the quality of the working-alliance was completed by 195 adult outpatients from the original sample (N = 212) (mean age of 39.6 years) (SD 11.4), who participated in this study. A majority of participants were male (70 %) and single (64 %); 21 % were divorced or widowed, and 15 % were married. Most were Dutch natives (62 %); 78 % had completed education to a moderate to high level. Moderate education level included high school and vocational college; a high education level consisted of further or higher education.

The commonest diagnosis was a psychotic disorder (81 %). The mean score on the log-transformed insight scale was 0.94 (SD 0.21), indicating average to high insight into one’s own illness. The mean score on the HoNOS was 11 (SD 5), which is consistent with the average score in psychotic patient populations (Mulder et al. 2004). The mean subscale scores on the HoNOS were as follows: 1.72 (SD 1.53) for behavioral problems, indicating mild behavioral problems; 2.09 (SD 1.37) for impairment, indicating mild cognitive and disability problems; 3.65 (SD 2.23) for symptoms, indicating moderate symptom severity; and 3.46 (SD 2.17) for social problems, indicating moderate severity of problems with regard to social relationships. The mean score on the locus of control scale was 14.45 (SD 4.69), indicating moderate perceived control about the events and ongoing life situations. The mean score on the social-support questionnaire was 41.89 (SD 9.10), indicating satisfaction about help received from family and friends.

Of the 101 participating clinicians, 56 % were female, 50 % were psychiatric nurses, 19 % were nurses, 7 % social workers, 6 % psychologists, 6 % residents in psychiatry and 2 % psychiatrists. Their average (median) working experience was 14 years, with a range from 1 to 35 years. Most clinicians rated the working alliance questionnaire on one patient. The number of ratings ranged between 1 and 9 patients.

Table 1 shows the total and subscale scores on patient- and clinician-rated WAI. The scores of the two versions were high, indicating satisfaction with the alliance. Patients were slightly more positive about their working alliance than their clinicians were. The paired *t* tests showed small but significant differences between the total patient and clinician WAI scales. The concordance between patient and clinician working alliance was low, with correlations ranging from 0.22 to 0.28.

**Table 1:** Correlations and paired *t* tests of the total and subscales scores of working-alliance inventory (WAI) from patients’ and clinicians’ perspectives

	WAI patient			WAI clinician			Correlation		Differences	
	N	Mean	SD	N	Mean	SD	*r*	*p*	*t*	*p*
Total WAI	195	142.97	22.24	195	137.82	14.90	0.28	0.00	3.12	0.00
Bond subscale	195	50.70	7.40	195	48.79	4.66	0.24	0.00	−3.43	0.00
Tasks subscale	195	47.09	8.09	195	45.76	5.52	0.28	0.00	−2.92	0.01
Goals subscale	195	45.18	8.36	195	43.27	6.02	0.22	0.00	−2.21	0.03

Table 2 presents the final multiple regression analysis. Hypothesis 1—that a higher level of psychosocial functioning would be associated with a clinician’s and patient’s perception of a better working alliance—was partly confirmed: patient-rated WAI was associated with (1) higher scores on the perceived treatment needs of the insight subscale, (2) lower HoNOS behavioral and social problem scores, and (3) higher social-support scores. The clinician-rated WAI was associated with higher level of illness awareness, and higher social support scores. But, contrary to our hypothesis, the patient-rated WAI was associated with severer psychiatric symptoms as assessed with the HoNOS. With regard to the second hypothesis—that the working alliance achieved with patients with an external locus of control would be perceived more poorly by clinician and patient—we found that locus of control was negatively associated with higher scores on the patient-rated WAI. This means that patients with greater control over their lives were more positive about their working alliance.

**Table 2:** Multiple regression analysis of the patient and clinician working-alliance inventory (WAI) and independent predictors

Variable	WAI patient			WAI clinician		
	*B*	SE *B*	p	*B*	SE *B*	p
Constant	115.01	10.34	0.00	143.11	6.91	0.00
Insight						
*Need for treatment*	20.96	6.46	0.00			
*Illness awareness*				7.97	4.06	0.05
Psychosocial functioning						
*Behavioural problems*	−2.52	1.01	0.01			
*Symptoms*	2.20	0.78	0.00			
*Social problems*	−1.36	0.79	0.09	−1.16	0.47	0.01
Social support	0.34	0.16	0.04			
Locus of control	−0.97	0.36	0.01			
Ethnicity (immigrants)	8.47	3.07	0.01			
Marital status (not married)				−6.27	2.90	0.03
Gender (women)				4.91	2.22	0.03

Patient WAI R^2^ = 0.20; therapist WAI R^2^ = 0.12

Finally, unlike the Dutch patients, immigrant patients scored higher on the patient-rated WAI. Married patients and women scored higher on the clinician-rated WAI. The final models accounted for 20 % of the total variance in the patient-rated WAI scores, and 12 % of the total variance in the clinician-rated WAI-scores.

## Discussion

This study in patients with psychotic and bipolar disorders investigated whether greater illness insight, better social functioning, fewer behavioral problems, and more social support were associated with more positive patients’ and clinicians’ ratings of their working relationship. We also examined whether the working alliance achieved with patients with an external locus of control would be perceived more poorly by the clinician and patient. As in previous studies (Barrowclough et al. 2010; Couture et al. 2006; Tryon et al. 2007; Wittorf et al. 2009), the correlation between clinicians’ and patients’ ratings of the working alliance was low to moderate, and patients qualified their alliance more positively than their clinicians did. In their meta-analyses, Tryon et al. (2007) report that the perceived working relationship between clinicians and patient shows more divergence for patients with less impairment and less psychiatric symptoms.

We found that the patients’ and clinicians’ perspectives on the working alliance were associated with different sets of variables. A working alliance that was rated positively by the patient was associated with more severe symptoms, a more strongly perceived need for treatment, fewer behavioral and social problems, being an immigrant, and an internal locus of control. Clinicians were more positive about the working alliance if a patient was married, female, had fewer social problems, and was more aware of his or her illness. Unlike the two studies that used only global assessments of insight (Barrowclough et al. 2010; Wittorf et al. 2009), our study differentiated several aspects of this variable.

Our finding that severer symptoms were associated with a better patient-rated working alliance was not consistent with previous studies. A possible explanation for this discrepancy is that Barrowclough et al. (2010) used positive and negative syndrome scales to measure symptomatology, while symptoms in our study comprised delusions, depressive mood and other symptoms. It is interesting that Barrowclough et al. (2010) did find a positive link between self-rated depression and alliance, because patients in our study scored high on the depressive mood subscale. It may be that depressive symptoms have a particularly pronounced relationship with the alliance. However, while McCabe and Priebe’s study (2003) also included depression in the measurement of the symptoms, it did not find any association with the alliance.

We also found that patients who experienced greater control over their lives were more positive about the working alliance with their therapist. Although it has not previously been studied in the context of working alliance, an internal locus of control was associated with increased treatment motivation, compliance and treatment adherence, and with better treatment outcomes in patients with severe mental illness (Harrow et al. 2009). Our findings may mean that a positive working alliance is an effect modifier for the link between locus of control and health behavior, and subsequent treatment outcomes.

Other researchers have studied why patients with severe mental illness may engage differently from patients with milder illnesses and how clinicians can better engage patients by modifying their approach to them (Freeman et al. 2013; Lavelle et al. 2014; McCabe et al. 2002). Based on these insights, interventions are being developed and tested that focus on structuring patient-clinician communication, and that routinely discuss the patient’s level of motivation for engaging in treatment (Jochems et al. 2012; Priebe et al. 2007, 2013). It seems likely that this testing will produce practical findings that are applicable to this population, since these interventions are designed especially to serve the needs of patients with severe mental illness.

Our study had five limitations. The first is that working alliance was measured through a self-report inventory of what patient and clinician thought of each other; no observer-rated assessment was included. The second limitation is that these variables explained only 20 % of the total variance in the patient-rated working alliance, and only 12 % of that in the clinician alliance. The variables that account for the unexplained part of the variance are unknown. The third limitation is that, due to the sample characteristics, generalization of the results is limited. Most participants were male, had a psychotic disorder, and, over a given period, had been in contact with Assertive Community treatment and Illness Management and Recovery teams. No information had been collected about how long these patients had been treated. The forth limitation is that the patients’ and clinicians’ perception of working alliance may vary according to the stage of the psychiatric illness or between mental healthcare settings. The final limitation is the cross-sectional nature of the study, which does not enable us to draw any causal conclusions.

With regard to clinical practice, our results suggest that patients and therapists may have different perceptions of the alliance. A possible implication of the finding that the implementation of crisis plans may be predicted by a clinician’s perspective on the alliance (Ruchlewska et al., submitted) is that clinicians need help in developing awareness of the goals and tasks of patients with certain characteristics—i.e., singles, men, and those with poorer social functioning and poorer insight into their illness and need for treatment—and in helping them effectively to consider them. The same finding may also mean that clinicians should become aware of a possible implicit preference for married female patients, for those who have a better understanding of their illness, and those are more competent in their social life. A focus on the patient’s own sense of responsibility for the treatment may also help to build a successful alliance. Future research should replicate the results of present study, and should also investigate whether patients’ treatment history, especially with their current clinician, would have an impact on their alliance.

## References

[CR1] Barrowclough C, Meier P, Beardmore R, Emseley R (2010). Predicting therapeutic alliance in clients with psychosis and substance misuse. Journal of Nervous and Mental Disease.

[CR2] Birchwood M, Smith J, Drury V, Healy J, Macmillan F, Slade M (1994). A self-report insight scale for psychosis: Reliability, validity and sensitivity to change. Acta Psychiatrica Scandinavica.

[CR3] Couture SM, Roberts DL, Penn DL, Cather C, Otto MW, Goff D (2006). Do basic client characteristics predict the therapeutic alliance in the treatment of schizophrenia?. Journal of Nervous and Mental Disease.

[CR4] Davis LW, Lysaker PH (2004). Neurocognitive correlates of therapeutic alliance in schizophrenia. Journal of Nervous and Mental Disease.

[CR5] Frank AF, Gunderson JG (1990). The role of the therapeutic alliance in the treatment of schizophrenia. Archives of General Psychology.

[CR6] Freeman D, Dunn G, Garety P, Weinman J, Kuipers E, Fowler D, Jolley S, Bebbington P (2013). Patients’ beliefs about the causes, persistence and control of psychotic experiences predict take-up of effective cognitive behavior therapy for psychosis. Psychological Medicine.

[CR7] Harrow M, Hansford BG, Astrachan-Kletcher EB (2009). Locus of control: Relation to schizophrenia, to recovery, and to depression and psychosis. Psychiatric Research.

[CR8] Henderson C, Flood C, Leese M, Thornicroft G, Sutherby K, Szmukler G (2004). Effect of joint crisis plans on use of compulsory treatment in psychiatry: Single blind randomized controlled trail. British Medical Journal.

[CR9] Henderson C, Swanson JW, Szmukler G, Thornicroft G, Zinkler M (2008). A typology of advance statements in mental health care. Psychiatric Services.

[CR10] Horvath AO, Greenberg LS (1989). Development and validation of the working alliance inventory. Journal of Counseling Psychology.

[CR11] Horvath AO, Symonds BD (1991). Relationship between working alliance and outcome in psychotherapy: A meta-analysis. Journal of Counseling Psychology.

[CR12] Jochems EC, Mulder CL, van Dam A, Duivenvoorden HJ, Scheffer SC, van der Spek W, van der Feltz-Cornelis CM (2012). Motivation and treatment engagement intervention trial (MotivaTe-IT): The effects of motivation feedback to clinicians on treatment engagement in patients with severe mental illness. BMC Psychiatry.

[CR13] Kempen GIJM (1992). Psychometric properties of GLAS baseline measures: A pilot study (in Dutch).

[CR14] Lavelle M, Dimic S, Wildgrube C, McCabe R, Priebe S (2014). Non-verbal communication in meetings of psychiatrists and patients with schizophrenia. Acta Psychiatrica Scandinavica.

[CR15] Lysaker PH, Davis LW, Buck KD, Outcalt S, Ringer JM (2011). Negative symptoms and poor insight as predictor of the similarity between client and therapist ratings of therapeutic alliance in cognitive behavior therapy for patients with schizophrenia. Journal of Nervous and Mental Disease.

[CR16] Martin DJ, Garske JP, Davis MK (2000). Relation of the therapeutic alliance with outcome and other variables: A meta-analytic review. Journal of Consulting and Clinical Psychology.

[CR17] McCabe R, Heath C, Burns T, Priebe S (2002). Engagement of patients with psychosis in the consultation: Conversation analytic study. British Medical Journal.

[CR18] McCabe R, Priebe S (2003). Are therapeutic relationships in psychiatry explained by patients’ symptoms? Factors influencing patient ratings. European Psychiatry.

[CR19] Mulder CL, Staring ABP, Loos J, Buwalda V, Kuijpers D, Sytema S, Wierdsma AI (2004). De Health of the Nations Outcome Scales in Nederlandse vertaling. Psychometrische kenmerken. Tijdschrift voor Psychiatrie.

[CR20] Pearlin LI, Schooler C (1978). The structure of coping. Journal of Health and Social Behavior.

[CR21] Priebe S, Kelley L, Golden E, McCrone P, Kingdon D, Rutterford C, McCabe R (2013). Effectiveness of structured patient-clinician communication with a solution-focused approach (DIALOG+) in community treatment of patients with psychosis, a cluster randomized controlled trial. BMC Psychiatry.

[CR22] Priebe S, McCabe R, Bullekamp J, Hansson L, Lauber C, Martinez-Leal R, Rössler W, Salize H, Svensson B, Torres-Gonzalves F, van den Brink R, Wiersma D, Wright DJ (2007). Structured patient-clinician communication and 1-year outcome in community mental health care: Cluster randomized controlled trial. British Journal of Psychiatry.

[CR23] Ruchlewska, A., Kamperman, A. M., Wierdsma, A. I., Van der Gaag, M., & Mulder, C. L. (submitted). Determinants of implementation and use of psychiatric advance statements in mental healthcare. *Psychiatric Services*. 10.1176/appi.ps.201400495 27079985

[CR24] Ruchlewska A, Mulder CL, Smulders R, Roosenschoon BJ, Koopmans G, Wierdsma AI (2009). The effects of crisis plans for patients with psychotic and bipolar disorders: A randomized controlled trial. BMC Psychiatry.

[CR25] Ruchlewska A, Wierdsma AI, Kamperman AM, Van der Gaag M, Smulders R, Roosenschoon B, Mulder CL (2014). Effect of crisis plans on admissions and emergency visits: A randomized controlled trial. PLoS One.

[CR26] Ruehlman LS, Lanyon RI, Karoly P (1999). Development and validation of the multidimensional health profile, part I: Psychosocial functioning. Psychological Assessment.

[CR27] Tryon GS, Collins-Blackwell S, Felleman-Hammel E (2007). A meta-analytic examination of client-therapist perspectives of the working alliance. Psychotherapy Research.

[CR28] Vervaeke GAC, Vertommen H (1996). De werkalliantievragenlijst (WAV). Gedragstherapie.

[CR29] Wing JK, Beevor AS, Curtis RH (1998). Health of the Nation Outcome Scale (HONOS): Research and development. British Journal of Psychiatry.

[CR30] Wittorf A, Jakobi U, Bechdolf A, Müller B, Sartory G, Wagner M, Wiedemann G, Herrlich J, Buchkremer G, Klingberg S (2009). The influence of baseline symptoms and insight on the therapeutic alliance early in the treatment of schizophrenia. European Psychiatry.

